# Risk for acquired coronary artery disease in genetic vs. congenital thoracic aortopathy

**DOI:** 10.3389/fcvm.2022.1036522

**Published:** 2023-01-12

**Authors:** Onur B. Dolmaci, Tugay Ayyildiz, Robert E. Poelmann, Antoine H. G. Driessen, Dave R. Koolbergen, Robert J. M. Klautz, Jan H. N. Lindeman, Nimrat Grewal

**Affiliations:** ^1^Department of Cardiothoracic Surgery, Leiden University Medical Center (LUMC), Leiden, Netherlands; ^2^Department of Cardiothoracic Surgery, Amsterdam University Medical Center, Amsterdam, Netherlands; ^3^Institute of Biology, Animal Sciences and Health, Leiden University, Leiden, Netherlands; ^4^Department of Cardiology, Leiden University Medical Center, Leiden, Netherlands; ^5^Department of Vascular Surgery, Leiden University Medical Center (LUMC), Leiden, Netherlands; ^6^Department of Anatomy and Embryology, Leiden University Medical Center, Leiden, Netherlands

**Keywords:** aortic dilatation, coronary artery disease, bicuspid aortic valve, Marfan syndrome, ascending aortic aneurysm

## Abstract

**Objective:**

Patients with Marfan syndrome (MFS) and patients with a bicuspid aortic valve (BAV) have a significantly increased risk to develop thoracic aortopathy. Both conditions share many pathophysiological mechanisms leading to aortic complications. Bicuspidy is known to have a low risk for acquired coronary artery sclerosis. The aim of this study is to determine the risk of coronary sclerosis in MFS patients.

**Methods:**

Marfan syndrome patients with an aortic root dilatation, which were surgically treated between 1999 and 2017, were included and matched with BAV and tricuspid aortic valves (TAV) patients based on sex and age. Cardiovascular risk profiles were determined in all three groups. Coronary sclerosis was graded in all patients on coronary imaging (coronary angiography or computed tomography) using a coronary artery scoring method, which divides the coronaries in 28 segments and scores non-obstructive (20–49% sclerosis) and obstructive coronary sclerosis (>49% sclerosis) in each segment.

**Results:**

A total of 90 matched patients (30 within each group) were included. MFS patients showed less cardiovascular risk factors compared to BAV and TAV patients. TAV patients had higher amounts of obstructive coronary sclerosis as compared to BAV patients (*p* = 0.039) and MFS patients (*p* = 0.032). No difference in non- and obstructive coronary artery disease (CAD) was found between the MFS and BAV population.

**Conclusion:**

Marfan syndrome and bicuspid aortic valve patients have a significantly lower risk for, and prevalence of CAD as compared to TAV individuals.

## 1. Introduction

Marfan syndrome (MFS) is one of the most common hereditary connective tissue disorders with a prevalence of 20 per 100.000, caused by a mutation in the fibrillin-1 gene (1). A bicuspid aortic valve (BAV) is the most common cardiac congenital anomaly, with a population prevalence of 1–2% (2, 3). Despite distinct underlying etiologies, both conditions share an extreme high risk to develop thoracic aortopathy as compared to patients with a tricuspid aortic valve (TAV) (4–6). Recent studies have aimed to unravel common pathogenetic mechanisms in both conditions which could lead to similar aortic complications. The findings revealed that bicuspid aortopathy has significantly less degenerative pathologic features such as inflammation, cytolytic necrosis and elastic fiber degeneration (7–10) as compared to MFS and TAV patients (11). The aortic media of BAV patients is characterized by a phenotypic switch defect of vascular smooth muscle cells in the ascending aorta, leading to a significantly lower amount of contractile smooth muscle cells (11). Furthermore, the intimal layer is embryologically altered resulting in a significantly thinner intima, with minimal features of atherosclerosis as compared to patients with a TAV (11–13). In line with the histopathological findings of less atherosclerosis, clinically the BAV patients have also demonstrated a significantly lower risk for coronary artery sclerosis (14–16).

Marfan syndrome patients have many ascending aortic wall features in common with the BAV population, including a phenotypic switch defect of the vascular smooth muscle cells as well as a significantly thinner intimal layer (7). In contrast to the BAV, however, degenerative pathologic features are also highly characteristic of the MFS patient’s aortic media, comparable with the TAV (7, 8, 17). Because MFS shows features of both BAV disease as well as degenerative thoracic aortopathy, we aimed to investigate the risk of acquired CAD in MFS patients. In this study we compare the cardiovascular risk profile and amount of coronary sclerosis in MFS, BAV, and TAV patients undergoing aortic root or valve replacement.

## 2. Materials and methods

### 2.1. Study population

Patients were included from two academic hospitals in the Netherlands: the Leiden University Medical Center and the Amsterdam University Medical Center. MFS patients were included from both hospitals (*n* = 197) and were all surgically treated (or intended to be) for an aortic root dilatation between 1999 and 2017. BAV and TAV patients (*n* = 87 and *n* = 152, respectively) were all included from the LUMC and were all operated due to an aortic regurgitation and/or aortic root dilatation between 2006 and 2020. Patients under the age of 18, patients with an active endocarditis and patients with an aortic dissection were excluded. Patients from the three groups (MFS, BAV, and TAV) were matched based on sex and age (±2 years), which resulted in 30 patients in each group. Patients with connective tissue disorders were excluded from the BAV and TAV groups. Electronic health records were searched to gather data on demographics, presence of common gene mutations for aortic disease/connective tissue disorders (e.g., FBN1, TGFB2 mutations), cardiovascular disease and risk profiles and surgical data. The aortic valve morphology was based on the surgical findings and for the BAV patients noted according to the Sievers classification (18). The medical ethics committees of the Leiden University Medical Center and Amsterdam University Medical Center both granted an approval for this study, patient consent was waived.

### 2.2. Coronary artery disease

The medical history of each patient was searched to identify previous coronary artery disease (CAD) events (e.g., myocardial infarction or angina and previous coronary revascularization) and CAD risk factors [i.e., a family history of CAD (aged younger than 65), hypertension, diabetes mellitus, and the body mass index] (19). Hypertension was scored if reported in a patient’s medical history, in case of active pharmacological treatment or when a blood pressure of ≥ 140 mmHg was reported on multiple occasions. Hypercholesterolemia was scored if the patient had a total cholesterol level of ≥ 6.5 mmol/l or used lipid-lowering medications. Diabetes was scored in cases with either a blood glucose level of ≥ 7.0 mmol/l on two separate (fasted) occasions, a glucose level of ≥ 11.1 mmol/l plus symptoms of hyperglycemia, or use of anti-diabetic medication.

**FIGURE 1:**
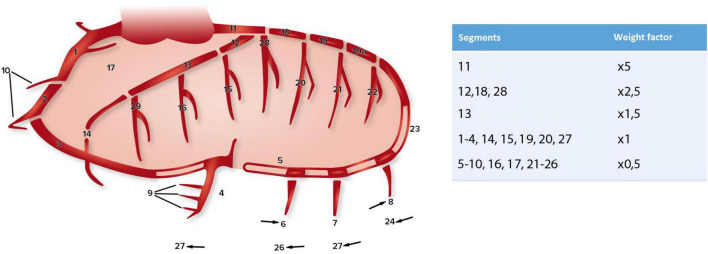
Coronary artery segments (according to CASS) and the corresponding weight factors used for the CAGE score, adapted from Scanlon et al. (20), Vlietstra et al. (21), and Emond et al. (22).

The severity of coronary artery sclerosis was determined based on preoperative coronary angiographies or computed tomographies. The CAGE score was used to score coronary sclerosis. This scoring system scores non-obstructive (CAGE ≥ 20, coronary sclerosis of 20–49%) and obstructive (CAGE ≥ 50, coronary sclerosis of ≥ 50%) sclerosis in 28 coronary segments (Figure 1) (15, 20–22). Weight factors (as shown in Figure 1) are used afterward to discriminate between important (proximal) and less important (distal) lesions. A computed tomography of the coronary arteries was used in those cases where a coronary angiography was not performed (*n* = 19). The coronary angiographies and/or computed tomographies were scored by two researchers independently. No systematic differences or errors were seen between the two methods and/or investigators within this study.

### 2.3. Statistical analysis

Descriptive continuous data are presented as a mean ± standard deviation or as median and interquartile range depending on the distribution. Categorical data are presented as frequencies and percentages. A normality test (Shapiro-Wilk test), kurtosis and skewness was performed for all variables. To analyze the differences between the three groups, an ANOVA (for continuous normally distributed data), Mann-Whitney U (continuous not normally distributed data) and/or a chi-square test (categorical data) was used.

For the relationship between aortic valve morphology and CAD, the ANOVA test with a *post hoc* procedure was used. A *post hoc* procedure was performed to identify statistical significance between the groups when significant differences were found. A *p*-value < 0.05 was considered statistically significant using SPSS 25.0 (SPSS Inc., Chicago, USA).

## 3. Results

A total of 90 matched patients were included with 30 patients in each group (MFS, BAV, and TAV). Tables 1, 2 display all baseline and surgical characteristics, respectively. Included patients were predominantly males (86.7%) with a mean age of 53 ± 9.1 SD. Of the MFS patients, 5 (16.7%) had a BAV and 25 (83.3%) had a TAV.

**TABLE 1:** Baseline characteristics.

Characteristics	MFS (*n* = 30)	BAV (*n* = 30)	TAV (*n* = 30)	*P*-value
**Sex**				
Male	26 (86.7)	26 (86.7)	26 (86.7)	1.000
Female	4 (13.3)	4 (13.3)	4 (13.3)	1.000
Age*	52.1 ± 8.4	54.8 ± 9.3	54.2 ± 9.7	0.510
Genetic mutations		-	-	
FBN1	13 (43.3)			
Other	3 (10)			
Unknown	14 (46.7)			
Body mass index	24.7 ± 3.0	25.9 ± 5.0	25.6 ± 3.9	0.486
Family history CAD	2 (6.7)	3 (10.0)	4 (13.3)	0.664
Diabetes mellitus	2 (6.7)	-	3 (10.0)	0.233
Hypertension	8 (26.7)	18 (60.0)	19 (63.3)	0.006
Hypercholesterolemia	-	6 (20.0)	8 (26.7)	0.044
Previous MI (NSTEMI/STEMI)	1 (3.3)	-	1 (3.3)	0.664
Pre-operative creatinine	74.2 ± 11.6	79.7 ± 13.4	82 (IQR 76.5–95.5)	0.045
**Imaging modality**				
CT	9 (30.0%)	4 (13.3%)	6 (20.0%)	0.289
CAG	21 (70.0%)	26 (86.7%)	24 (80.0%)	0.289

*Age at the time of coronary imaging. Data are presented as *N* (%), mean ± SD or median (interquartile range).

BAV, bicuspid aortic valve; CAD, coronary artery disease; CAG, coronary angiography; CT, computed tomography; FBN1, fibrillin-1; MFS, Marfan syndrome; MI, myocardial infarction; MVP, mitral valve plasty; MVR, mitral valve replacement; NSTEMI, non-ST-elevation myocardial infarction; STEMI, ST-elevation myocardial infarction; TAV, tricuspid aortic valve; TVP, tricuspid valve plasty; VSRR, valve sparing root replacement.

**TABLE 2:** Surgical characteristics.

Characteristic	MFS (*n* = 30)	BAV (*n* = 30)	TAV (*n* = 30)	*P*-value
VSRR	26 (86.7)	-	-	<0.001
AVR + aortic surgery	-	21 (70)	13 (43.3)	<0.001
Isolated AVR	-	9 (30)	17 (57.7)	0.041
**Concomitant procedures**				
CABG	2 (6.7)	2 (6.7)	9 (30.0)	0.016
MVP	6 (20)	1 (3.3)	4 (13.3)	0.100
MVR	-	-	2 (6.7)	0.143
TVP	2 (6.7)	-	4 (13.3)	0.124
Rhythm surgery	1 (3.3)	2 (6.7)	3 (10.0)	0.644

Number of patients for whom data was available. Data are presented as *N* (%).

AVR, aortic valve replacement; BAV, bicuspid aortic valve; CABG, coronary artery bypass grafting; MFS, Marfan syndrome; MVP, mitral valve plasty; MVR, mitral valve replacement; TAV, tricuspid aortic valve; TVP, tricuspid valve plasty; VSRR, valve sparing root replacement.

### 3.1. Cardiovascular (risk) profiles

**FIGURE 2:**
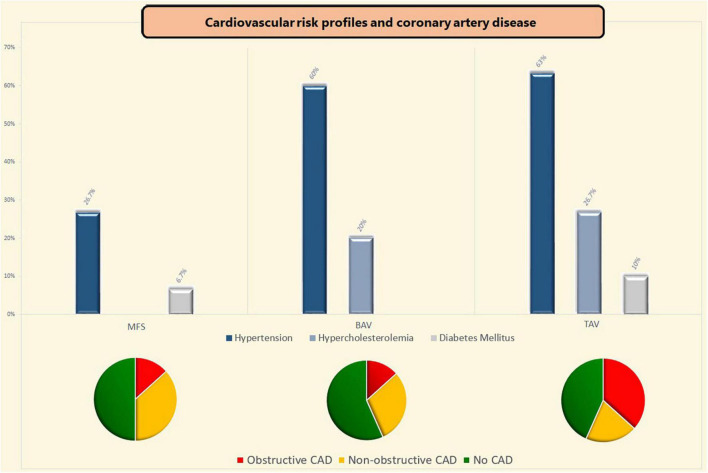
Coronary artery disease and cardiovascular risk profiles. BAV, bicuspid aortic valve; CAD, coronary artery disease; MFS, Marfan syndrome; TAV, tricuspid aortic valve.

Comparison of CAD risk factors showed a lower incidence of hypertension in MFS patients compared to BAV [OR 4.13 (95% CI 1.39–12.27); *p* = 0.023] and TAV patients [OR 4.75 (95%CI 1.58–14.25); *p* = 0.011]. Hypercholesterolemia was also less prevalent in MFS patients when compared to TAV patients [OR 3.25 (95%CI 1.11–9.52); *p* = 0.040], but did not differ significantly from BAV patients (*p* = 0.187) (see Figure 2).

Of the included patients, one within the MFS group (3.3%) and one patient in the TAV group (3.3%) had a history of myocardial infarction (*p* = 0.664). The previous myocardial infarction, which is an uncommon finding within the MFS group, was seen in a male patient of 51-years old who suffered a silent myocardial infarction [with non-significant coronary sclerosis (up to 40%) on the preoperative coronary angiography]. Concomitant coronary artery bypass grafting due to significant coronary artery sclerosis at the time of surgery was more often performed in TAV patients as compared to both MFS [OR 5.36 (95%CI 1.04–27.57); *p* = 0.053] and BAV patients [OR 6.00 (95%CI 1.18–30.73); *p* = 0.037]. Two MFS patients received concomitant coronary artery bypass grafting because of periprocedural coronary button defects (neo left coronary artery and right coronary artery—aortic anastomosis). Table 3 shows the results of the comparisons of the preoperative coronary imaging between all three groups. Non-obstructive coronary sclerosis (20–49% coronary obstruction) was not significantly different between the three groups (*p* = 0.499). Obstructive coronary sclerosis (≥50% coronary obstruction) was less prevalent in MFS and BAV patients as compared to TAV patients (*p* = 0.032 and *p* = 0.039, respectively, see Table 3). Coronary sclerosis did not differ between BAV and MFS patients (CAGE ≥ 20; *p* = 0.428 and CAGE ≥ 50; *p* = 1.000). The effects of each CAD risk factor on the CAGE scores were calculated per group and were non-significant in all cases.

**TABLE 3:** Comparisons of obstructive coronary sclerosis.

Comparison	CAGE ≥ 50 scores*	*P*-value^†^	Odds ratio (95% CI)	*P*-value^†⁣†^
MFS vs. TAV	0.00 (0.00–0.00) vs. 0.00 (0.00–2.5)	0.032	0.64 (0.41–0.99)	0.049
MFS vs. BAV	0.00 (0.00–0.00) vs. 0.00 (0.00–0.00)	1.000	1.08 (0.71–1.66)	0.710
BAV vs. TAV	0.00 (0.00–0.00) vs. 0.00 (0.00–2.5)	0.039	0.76 (0.54–1.07)	0.114

*Medians (interquartile range) are presented in the same order as the presented in the comparison column.

^†^Mann-Whitney *U*-test.

^†⁣†^Regression analysis.

Data are presented as mean ± SD. BAV, bicuspid aortic valve; MFS, Marfan’s syndrome; TAV, tricuspid aortic valve.

## 4. Discussion

In the present study we found a lower prevalence of cardiovascular risk factors and coronary sclerosis in patients with MFS and thoracic aortopathy as compared to patients with a TAV. Coronary sclerosis was studied directly on coronary angiographies and scored according to the CAGE score system in which (non) obstructive lesions are scored in 28 different segments of the coronary arteries. Our results showed a lower amount of CAD (lower CAGE scores) in patients with a BAV and the MFS group compared to TAV patients. The results of this study are consistent with our prior fundamental impression that aortic aneurysm formation in genetic and congenital diseases is associated with a lower prevalence of systemic atherosclerosis. Clinically thoracic aortic calcification has earlier been studied to assess the amount of systemic atherosclerosis in thoracic aortopathy (16). Although being a late marker of atherosclerosis, calcification was found less apparent in the aortopathy cases in that study independent of the major cardiovascular risk factors. Underlying genetic or congenital diseases were, however, not studied separately (16).

Patients with a BAV and patients with MFS have an extremely high risk to develop a thoracic aortic aneurysm and/or dissection at a much younger age as compared to patients with a TAV (23). Age is an important factor in the atherosclerotic disease process and is related with a significant change in the physiological and pathological properties of the vessel wall (24). Nevertheless, our study emphasized that in patients with a TAV and degenerative thoracic aortopathy, in the presence of cardiovascular risk factors, extensive systemic atherosclerosis can occur at a young age too. On the other hand in MFS and BAV the prevalence of cardiovascular risk factors and level of coronary sclerosis were significantly lower at the same age, despite having a thoracic aortic aneurysm in most. It has earlier been postulated that thoracic aortic aneurysms act protective for the development of systemic atherosclerosis (16). We hypothesize that a phenotypic switch defect which is similar in MFS and BAV individuals might play a role in the decreased risk for atherosclerosis.

Atherosclerosis is a chronic systemic disease that affects the arterial system and causes hardening and occlusion of arteries through plaque formation (25). These atherosclerotic plaques usually consist of a combination of cells (including smooth muscle cells, macrophages, and leucocytes), lipid deposits and extracellular matrix (25, 26). Plaques typically develop in the intimal layer of the aortic wall with also interference of the medial layer through the migration and proliferation of vascular smooth muscle cells to the intimal layer (27). This is particularly interesting, since both MFS and BAV patients have a significantly thinner intimal layer as compared to TAV patients. Another common feature between MFS and BAV patients is the aortic wall immaturity, e.g., the phenotypic switch defect of vascular smooth muscle cells. A primary function of vascular smooth muscle cells is contraction, in normal conditions, they exhibit extensive phenotypic diversity and plasticity (28). In pathological conditions, a switch can occur from a contractile to synthetic phenotype (29). Both MFS and BAV patients are known with a phenotypic switch defect, with a high expression of “undifferentiated” synthetic vascular smooth muscle cells. The synthetic phenotype of the smooth muscle cells is prone for migration and proliferation and the development of atherosclerotic plaques (30–33). As this is not seen in the MFS and BAV we hypothesize that the thin intimal layer in both patient groups might “protect” against plaque formation by complicating the migration and proliferation of vascular smooth muscle cells to the intimal layer. Future histopathological studies should focus on the migratory and proliferative effects of vascular smooth muscle cells into the intima in MFS and BAV.

### 4.1. Limitations

Marfan syndrome and bicuspid aortic valve patients develop aortopathy and/or aortic valve dysfunction generally at a younger age when compared to TAV patients. This could cause a selection bias, however, by matching the three groups based on age and gender these chances are minimalized. Moreover, we chose to include patients with end stage thoracic aortopathy referred for surgery, as these patients are likely to present with higher cardiovascular risk profiles. A small portion of the MFS patients had a BAV morphology (16.7%) which could influence the results, although the expected influence is small since the bigger part of the MFS group had a TAV morphology. Only thirty patients could be included in each group due to the rarity of the included syndromes and therefore small surgical population. The small sample sizes may have influenced the comparisons.

## 5. Conclusion

Marfan syndrome and bicuspid aortic valve patients have a significantly lower risk for, and prevalence of CAD as compared to TAV individuals. A common pathogenetic mechanism including a phenotypic switch defect and a thin intimal layer might underly the reduced risk for systemic atherosclerosis.

## Data availability statement

The raw data supporting the conclusions of this article are available upon reasonable request.

## Author contributions

JL, NG, and OD conceived and planned the manuscript, carried out analysis of the results, and wrote the manuscript. OD and TA scored the angiographies. NG was responsible for the overall content as guarantor. All authors reviewed and edited the manuscript.
